# Study on the relationship between NF-kB pathway and skeletal muscle dopamine receptors in muscle-attenuated mice

**DOI:** 10.4314/ahs.v24i3.48

**Published:** 2024-09

**Authors:** Hui Tian, Xinyu Wang, Shanshan Wu, Fengling Yang, Yue Han

**Affiliations:** 1 Department of General Practice, The First Affiliated Hospital of Harbin Medical University, Harbin, China; 2 Department of Gerontology, The First Affiliated Hospital of Harbin Medical University, Harbin, China

**Keywords:** Nuclear factor kB signaling pathway, Muscle attenuation, Mice, Skeletal muscle, Dopamine receptor

## Abstract

**Background:**

To investigate the relationship between the NF-kB signaling pathway and muscle-attenuated skeletal muscle dopamine receptor (DR) in mice.

**Methodology:**

40 specific pathogens free (SPF) C57BL/6 mice aged 6 to 7 months were randomly divided into a model group and control group by random number table method, with 20 mice in each group. Muscle-attenuated mice were established in model group, and the expression of NF-kB protein was detected by Western-blot, and the expressions of IL-β1, TNF-a, DRD1, and DRD2 were detected by enzyme linked immunosorbent assay (ELISA). Meanwhile, the muscle fiber cross-sectional area was determined.

**Results:**

The relative expression of NF-kB protein, IL-,1 and TNF-a in model group were significantly higher than those in control group (P < 0.05). The levels of DRD1 and DRD2 in skeletal muscle in model group were significantly lower than those in control group (P < 0.05). The cross-sectional are the model group was significantly lower than in the control group (P < 0.05). The relative expression of NF-kB protein was negatively correlated with DRD1, DRD2 and muscle fiber cross-sectional area. DRD1 and DRD2 were positively correlated with muscle fiber cross-sectional area.

**Conclusion:**

In muscle-atrophied mice, NF-kB protein, an indicator of the NF-kB signaling pathway, was negatively correlated with DR, and both had an important role in muscle atrophy.

## Introduction

Muscle decay syndrome refers to a syndrome in which skeletal muscle mass and muscle strength and function decrease 1,2. Anything that causes an increase in muscle breakdown and a decrease in muscle synthesis can lead to muscle decay syndrome, which can be caused by inflammation, oxidative stress, drug and hormone imbalances, and more 3. Researchers often study muscle decay syndrome by building animal models 4. Literature report 5 that dexamethasone can successfully construct a mouse model of muscle decay syndrome. In order to find ways to improve muscle decay syndrome, it is necessary to understand the biological signaling pathways and factors associated with the disease 6. At present, changes in muscle mass, muscle strength, and fiber cross-sectional area are often used to diagnose and evaluate muscle attenuation synthesis 7,8. The detection methods of the above evaluation indicators are complicated and lack of predictability, and more sensitive and predictable indicators are constantly sought in clinical practice. The regulatory function of NF-κB is reflected in both inflammatory and autoimmune reactions, and the abnormal expression level of NF-κB may affect the body's defense ability and cause tissue damage 9. NF-κB plays a vital role in the development of idiopathic inflammatory myopathy, and is also considered to play a role in muscle attenuation 10. Dopamine binds to its receptors and has a significant impact on motor function, the study said 11. In this study, dopamine receptor D1 (DRD1) and dopamine receptor D2 (DRD2) were considered to be closely related to the occurrence of muscle attenuation. There are few studies on the relationship between NF-κB pathway and skeletal muscle dopamine receptors in muscle-attenuated mice.

## Materials and methods

### Experimental animals and grouping

Forty SPF C57BL/6 mice aged from 6 to 7 months were selected and purchased from Beijing Weitonglihua Experimental Animal Technology Co., LTD. (Beijing, China). The certificate number of experimental animal was SCXK (Beijing) 2012-0001. Mice were acclimatized and fed for 1 week at a room temperature of 21-25°C and a relative humidity of 45%-55%, alternating between day and night for 12 h, with free access to food and water. Mice were randomly divided into model group and control group by random number table method, with 20 mice in each group. Muscle attenuated mice were established in model group, and mice in control group were injected subcutaneously with 0.9% normal saline. During the experiment, 2 and 3 mice died in model and control groups, respectively, and 18 and 17 mice in the two groups were included in the study. The Animal Ethics Committee of Harbin Medical University Animal Center approved the study.

### Experimental methods

#### Muscle attenuation model was established

After weighing, body composition and grasping for 24 h, model group mice were subcutaneously injected with 0.5 mg/ml dexamethasone. Control group mice were subcutaneously injected with 0.9% normal saline at 0.01 ml/g/d, for 19 consecutive days. The mice were free to drink and eat during modelling. Methods and criteria of successful modelling included that the general state of mice was observed every day during modelling, mainly including mental state, hair color, activity, etc. Body weight and body composition were measured every 3 days for the first 2 weeks and every 7 days for the last 4 weeks. Swimming speed and falling times from the wheeled running platform were measured after 6 weeks. The muscle mass and function of the model group decreased significantly compared with the control group. There were 18 successful models in the model group. A control group of 17 was allowed for follow-up.

NF-kB signaling pathway-related factors were detected The mice were anesthetized by intraperitoneal injection of pentobarbital sodium at a dose of 30 mg/kg. 2 mL of abdominal aortic blood was taken from the mice and the serum was separated for testing.NF-B level was detected by Western blot.

The levels of IL-1β and TNF-α were detected by ELISA.

#### Dopamine receptor (DR) detection in skeletal muscle

The animals were decapitated, the cortical tissue was taken, PBS homogenate was added, and the supernatant was collected for testing. The levels of DRD1 and DRD2 were detected by ELISA.

#### Muscle fibcross-sectionion measurement

The intermediate muscle tissue of skeletal muscle of mice was taken according to the above anesthesia method and tissue section was conducted. The sections were stained with SDH cholinesterase (AchE) to measure the cross-sectional area of muscle fibers in the middle of skeletal muscle.

#### Statistical analysis

Statistical Product and Service Solutions (SPSS) 22.0 software (IBM, Armonk, NY, USA) was used for statistical analysis, measurement data were expressed by (x̅±s), and t-test was used for comparison. Correlation was analysed by Pearson correlation analysis. P < 0.05 was considered to be significant in comparison of inter-group indicators.

## Results

### Comparison of NF-kB signaling pathway related factors between model group and control group

The relative expression of NF-kB protein, IL-1B and TNF-a in model group were significantly higher than those in control group (P < 0.05). See Table 1.

**Table 1:** Comparison of NF-κB signaling pathway related factors between model group and control groupκ

group	Only number	Relative expression of NF-κB protein	Il-1β (pg/ml)	Tnf-α (pg/ml)
Model group	18	1.21±0.22	220.10±32.21	145.54±21.15
Control group	17	0.72±0.19	121.19±27.81	88.29±13.43
t		7.033	9.698	9.494
P		0.000	0.000	0.000

### Comparison of DRD1 and DRD2 in skeletal muscle of model group and control group

The levels of DRD1 and DRD2 in skeletal muscle in model group were significantly lower than those in control group (P < 0.05), as shown in Table 2.

**Table 2:** Comparison of DRD1 and DRD2 in skeletal muscle of model group and control group

group	Only number	DRD1 (mg/g)	DRD2 (mg/g)
Model group	18	91.02±20.43	82.27±18.28
Control group	17	115.39±19.27	101.23±21.19
t		-3.625	-2.839
P		0.001	0.008

### Comparison of muscle fiber area between model group and control group

**Figure 1:**
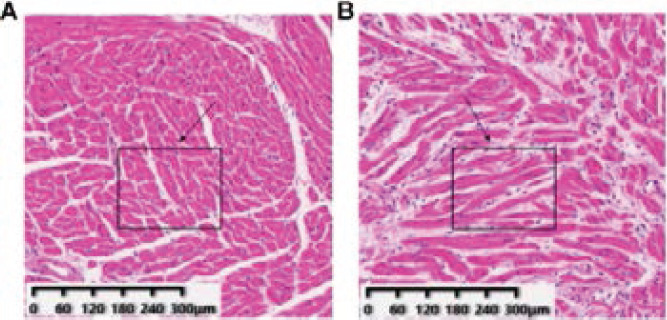
HE staining of muscle fiber area in model group and control group. (×200; (A) was the model group, (B) was the control group; Arrow shows measuring area)

The cross-sectional area of muscle fiber in model group was significantly lower than that in control group (P < 0.05), as shown in Table 3 and Figure 1.

**Table 3:** Comparison of muscle fiber area between model group and control group

group	Only number	Muscle fiber cross-sectional area (mm)^2^)	t	P
Model group	18	280.20±26.33	-27.605	0.000
Control group	17	540.40±29.42		

### Correlation analysis

Correlation analysis of NF-kB signaling pathway related factors, DRD1, DRD2 and muscle fiber cross-sectional area showed that the relative expression level of NF-kB protein was negatively correlated with DRD1, DRD2 and muscle fiber cross-sectional area (P < 0.05). DRD1 and DRD2 were positively correlated with muscle fiber cross-sectional area (P < 0.05). See Table 4.

**Table 4:** Correlation analysis results

index	Relative expression of NF-κB protein	IL-1β	TNF-α	DRD1	DRD2	Muscle fiber cross-sectional area
Relative expression of NF-KB protein	-	0.201	0.198	-0.322*	-0.304*	-0.441*
IL-1B	0.201	-	0.118	0.108	0.088	0.132
TNF-A	0.198	0.118	-	0.072	0.113	0.109
DRD1	-0.322*	0.108	0.072	-	0.112	0.401*
DRD2	-0.304*	0.088	0.113	0.112	-	0.367*
Muscle fiber cross-sectional area	-0.441*	0.132	0.109	0.401*	0.367*	-

Note:

* means P < 0.05.

## Discussion

Muscle decay is a syndrome characterized by reduced skeletal muscle mass, muscle strength, or muscle function associated with aging^12^. Clinical studies have shown that patients treated with dexamethasone have significantly reduced measured muscle content, so the drug is often used to establish animal models of muscle decay^13^. This study used dexamethasone to construct a muscle attenuation mouse model, and the levels of NF-kB signaling pathway-related factors were analysed. The results showed that the relative expression of NF-kB protein, IL-1β and TNF-α levels in model group was significantly higher than those in the control group. Studies have shown that^8^ muscle attenuation can inhibit the transmission of afferent signals from muscle receptors and inhibit the activity of motor neurons. Blood supply can affect the performance of muscles, reduce the supply of oxygen throughout the body, reduce the ability of the body cells to take oxygen and metabolism, resulting in abnormal immune system. However, immune abnormalities are usually closely related to the activation of the NF-κB pathway^14^. Activation of NF-κB signaling pathway leads to the transfer of NF-κB protein to the nucleus, and abnormally increases the expression of TNF-α, IL-1β, and other inflammatory factors^15^. In this study, the relative expression of NF-κB protein and the levels of IL-1β and TNF- α in the model group were significantly higher than those in the control group, further explaining the role of the above factors in the occurrence of muscle attenuation.

The results of this study showed that the levels of DRD1 and DRD2 in skeletal muscle of model group were significantly lower than those of control group. At present, studies have confirmed that the combination of dopamine with DRD1 and DRD2 in vertebrates is beneficial to the central nervous system to regulate motor rhythm. If the level of DRD1 and DRD2 is insufficient, motor atrophy will be caused. Literature report^16^ shows a complex relationship between motor neurons and skeletal muscle cells. The maintenance of normal morphological structure of muscle cells depends on the maintenance of related neural functions. The loss of DRD1 and DRD2 easily leads to metabolism and muscle morphological structure. Combined with the results of this study, DRD1 and DRD2 levels were correlated with nerve atrophy innervating skeletal muscle in muscle attenuation syndrome mice.

The results of this study showed that the muscle fiber cross-sectional area in model group was significantly lower than that in the control group. In muscle attenuated mice, muscle fiber is prone to fatigue and ATP regeneration obstacles. Muscles are often in a rigid state, which may lead to the mechanical destruction of muscle fibers, as shown by a reduction in the cross-sectional area of muscle fibers.

The results of this study showed that the relative expression level of NF-κB protein was negatively correlated with DRD1, DRD2, and muscle fiber cross-sectional area. DRD1 and DRD2 were positively correlated with muscle fiber cross-sectional area. In this study, muscle fibers of various types of skeletal muscle were atrophied after the establishment of the muscular attenuating disease mouse model. DRD1 and DRD2 are widely distributed in the cerebral cortex, which controls motor function^15^. Dysregulation or abnormality of reflex regulation in mice with muscular attenuating disease, the interaction between neurostimulator and inhibitory factor plays an important role in muscle attenuating disease^17^. One study claimed^18^ that when dopamine and its receptor levels are increased, it promotes neurotransmitter excitation and modulates the corticospinal gamma-reflex circuit, facilitating improved muscle tension and attenuation.

In conjunction with the results of this study, reduced levels of DRD1 and DRD2 result in increased muscle decay and tension, increasing the degree of muscle decay and reducing in muscle fiber cross-sectional area. The elevated relative expression of NF-κB protein in muscle-depleted mice affects the central pattern generator, which has a profound impact on muscle movement. There are abnormalities in the associated inflammatory factors, which can cause imbalances in anabolic and catabolic metabolism. Therefore, abnormally increased relative expression of NF-κB promotes muscle failure and decreases muscle fiber cross-sectional area.

In this study, we constructed a mouse model of muscle decay to elucidate some biochemical pathways of muscle decay syndrome to better understand the mechanism of muscle decay and develop interventions to prevent, delay, prevent and even reverse this phenomenon. The results of this study have specific clinical significance.

In conclusion, in muscle attenuation mice, NF-κB signaling pathway indicator NF-κB protein is negatively correlated with DR, and both of them play an essential role in muscle attenuation.

## Data Availability

The datasets used and analysed during the current study are available from the corresponding author on reasonable request.
